# Volume Estimates in Chronic Hemodialysis Patients by the Watson Equation and Bioimpedance Spectroscopy and the Impact on the Kt/V_urea_ calculation

**DOI:** 10.1177/2054358117750156

**Published:** 2018-01-10

**Authors:** Nazanin Noori, Ron Wald, Arti Sharma Parpia, Marc B. Goldstein

**Affiliations:** 1 Division of Nephrology, St. Michael’s Hospital, Toronto, Ontario, Canada; 2 Li Ka Shing Knowledge Institute, St. Michael’s Hospital, Toronto, Ontario, Canada

**Keywords:** bioimpedance spectroscopy, fat mass, hemodialysis, Kt/V, total body water, Watson formula

## Abstract

**Background:**

Accurate assessment of total body water (TBW) is essential for the evaluation of dialysis adequacy (Kt/V_urea_). The Watson formula, which is recommended for the calculation of TBW, was derived in healthy volunteers thereby leading to potentially inaccurate TBW estimates in maintenance hemodialysis recipients. Bioimpedance spectroscopy (BIS) may be a robust alternative for the measurement of TBW in hemodialysis recipients.

**Objectives:**

The primary objective of this study was to evaluate the accuracy of Watson formula–derived TBW estimates as compared with TBW measured with BIS. Second, we aimed to identify the anthropometric characteristics that are most likely to generate inaccuracy when using the Watson formula to calculate TBW. Finally, we derived novel anthropometric equations for the more accurate estimation of TBW.

**Design and Setting:**

This was a cross-sectional study of prevalent in-center HD patients at St Michael’s Hospital.

**Patients:**

One hundred eighty-four hemodialysis patients (109 men and 75 women) were evaluated in this study.

**Measurements:**

Anthropometric measurements including weight, height, waist circumference, midarm circumference, and 4-site skinfold (biceps, triceps, subscapular, and suprailiac) thickness were measured; fat mass was measured using the formula by Durnin and Womersley. We measured TBW by BIS using the Body Composition Monitor (Fresenius Medical Care, Bad Homburg, Germany).

**Methods:**

We used the Bland-Altman method to calculate the difference between the TBW derived from the Watson method and the BIS. To derive new equations for TBW estimation, Pearson’s correlation coefficients between BIS-TBW (the reference test) and other variables were examined. We used the least squares regression analysis to develop parsimonious equations to predict TBW.

**Results:**

TBW values based on the Watson method had a high correlation with BIS-TBW (correlation coefficients = 0.87 and *P* < .001). Despite the high correlation, the Watson formula overestimated TBW by 5.1 (4.5-5.8) liters and 3.8 (3.0-4.5) liters, in men and women, respectively. Higher fat mass and waist circumference (general and abdominal obesity) were correlated with the greater TBW overestimation by the Watson formula. We created separate equations for men and women based on weight and waist circumference.

**Limitations:**

The main limitation of our study was the lack of an external validation for our novel estimating equation. Furthermore, though BIS has been validated against traditional reference standards, our assumption that it represents the “gold standard” for body compartment assessment may be flawed.

**Conclusions:**

The Watson formula generally overestimates TBW in chronic dialysis recipients, particularly in patients with the highest waist circumference. Widespread reliance on the Watson formula for derivation of TBW may lead to the underestimation of Kt/V_urea._.

## Abrégé

**Contexte:**

Une évaluation précise du volume d’eau total (VET) de l’organisme est essentielle pour valider l’efficacité de la dialyse (Kt/V_urée_). Recommandée pour le calcul du VET, la formule de Watson a pourtant été établie en fonction de volontaires sains. Conséquemment, elle fournit des estimations potentiellement inexactes chez les patients hémodialysés. La spectroscopie de bio-impédance (BIS – Bioimpedance Spectroscopy) pourrait s’avérer une alternative fiable pour mesurer le VET des patients hémodialysés.

**Objectifs de l’étude:**

Notre principal objectif consistait à comparer l’exactitude des valeurs de VET mesurées par la formule de Watson et par bio-impédance. Secondairement, nous cherchions à cerner les caractéristiques anthropométriques les plus susceptibles d’engendrer des valeurs imprécises avec la formule de Watson. Enfin, nous voulions dériver des équations anthropométriques fiables pour mesurer le VET des patients.

**TYPE et CADRE de l’étude:**

Nous avons mené une étude transversale auprès de patients hémodialysés à l’hôpital St Micheal’s de Toronto.

**Patients:**

Un total de 184 patients (109 hommes et 75 femmes) ont participé à l’étude.

**Mesures:**

Ont été effectuées une série de mesures anthropométriques : poids, grandeur, tour de taille, périmètre brachial et épaisseur de quatre plis cutanés (au biceps, au triceps, sous l’omoplate et au niveau de l’iliaque supérieur). Ces données ont servi à calculer la masse adipeuse avec l’équation de Durnin et Womersley. Pour les mesures du VET par bio-impédance (BIS), on a utilisé un *Body Composition Monitor* ou BCM (Fresenius Medical Care, à Bad Homburg, en Allemagne).

**Méthodologie:**

Nous avons utilisé la méthode de Bland-Altman pour calculer l’écart entre les mesures de VET obtenues par la formule de Watson et par BIS. Pour guider l’élaboration d’équations plus fiables, on a calculé les coefficients de corrélation de Pearson la bio-impédance (test de référence) et d’autres variables. On a développé des équations simplifiées et concises permettant de prédire le VET avec la régression par les moindres carrés.

**Résultats:**

Les valeurs de VET obtenues par la formule de Watson se sont avérées très étroitement corrélées avec les valeurs obtenues par bio-impédance (coefficient de corrélation : 0,87; *p*<0,001). Toutefois, la formule de Watson a surévalué le VET de 5,1 litres en moyenne (entre 4,5 et 5,8 litres) chez les hommes et de 3,8 litres en moyenne (entre 3,0 et 4,5 litres) chez les femmes. Une masse adipeuse élevée et un fort tour de taille (cas d’obésité générale et d’obésité abdominale) ont été associés aux plus importantes surestimations du VET données par la formule de Watson. Nous avons dérivé des équations distinctes pour les hommes et les femmes en tenant compte du poids du patient et de son tour de taille.

**Limites de l’étude:**

L’absence de validation externe des nouvelles équations élaborées pour l’estimation du VET constitue la principale limite de notre étude. Par ailleurs, bien que la spectroscopie de bio-impédance ait été validée contre les étalons de référence conventionnels, notre supposition selon laquelle cette méthode représenterait l’étalon par excellence pour mesurer la composition corporelle pourrait être erronée.

**Conclusion:**

La formule de Watson surestime généralement le VET des patients hémodialysés, particulièrement chez ceux qui présentent un fort tour de taille. Ainsi, le recours généralisé à cette formule pour la dérivation du VET des patients hémodialysés pourrait mener à une sous-évaluation du Kt/V_urée_.

## What was known before

The total body water (TBW) estimation is a critical component of the estimation of dialysis adequacy. The formula derived by Watson et al which has been used for the calculation of TBW was derived in mostly healthy volunteers and is based on BMI, which does not differentiate between fat and muscle, which have different water contents.

## What this adds

We have shown that the Watson formula provides a gross overestimation of TBW in chronic hemodialysis recipients. Measurement of TBW using contemporary bioimpedance technology has the potential to mitigate this problem. Similarly, the estimation of TBW using our novel equations, (which consider the waist circumference as an important reflection of fat mass), if validated, might provide clinicians with a more refined assessment of TBW and thus help guide appropriate adjustments to the dialysis prescription.

## Introduction

Accurate assessment of total body water (TBW) is vital for the management of maintenance hemodialysis (HD) recipients. TBW reflects the volume of distribution of urea and constitutes the denominator of the Kt/V_urea_, the widely used marker of small molecule clearance in a given HD session.^1,2^ Accurate assessment of TBW is also needed for the online measurement of Kt/V_urea_ by sodium dialysance, a feature offered by many contemporary HD machines. The assessment of TBW has relied on a variety of equations, each with assumptions that may be inappropriate for the typical dialysis recipient. This issue is crucial as the accuracy and precision of TBW estimation has the potential to significantly influence the clinician’s perception of dialysis adequacy.

Several formulae have been derived for the estimation of TBW though only one of these was developed in a population of HD recipients.^3-5^ The formula derived by Watson et al,^5^ in which the key variables are the patient’s weight and height, has been endorsed by the Kidney Disease Outcomes Quality Initiative (KDOQI) Clinical Practice Guidelines for HD adequacy for the calculation of TBW.^2,5^ This equation was derived in mostly healthy volunteers, and although some were hospitalized for minor disorders, patients who had evidence of edema or conditions that could alter volume status were excluded.^5^ It has been shown that the body composition of the HD population is fundamentally different from that of the general population due to a variety of factors that influence TBW content including undernutrition, lower lean tissue mass, higher fat mass, and higher extracellular water.^6,7^ Thus, the appropriateness of applying the Watson formula to the dialysis population should be reevaluated.

The primary objective of this study was to evaluate the accuracy of Watson formula–derived TBW estimates as compared with TBW measured with bioimpedance spectroscopy (BIS). Second, we aimed to identify the anthropometric characteristics that are most likely to generate inaccuracy when using the Watson formula. Finally, we derived alternative anthropometric equations that may be more appropriate for a population of hemodialysis recipients.

## Materials and Methods

### Study Design

This is a cross-sectional study of prevalent in-center HD patients at St Michael’s Hospital, a tertiary care teaching hospital, in Toronto, Canada. Adults 18 years or older who had been receiving conventional maintenance HD for at least 3 months were eligible for this study. Exclusion criteria were pregnancy or limb amputation. All patients were receiving conventional HD (3-4 hours per session, 3-4 times weekly) at the time of assessment. The dialysis machine was the Fresenius 5008 (Fresenius Medical Care, Bad Homburg, Germany), and the predominant dialyzer was Fx CorDiax 120 (Fresenius Medical Care, Bad Homburg, Germany). We collected relevant demographic and clinical data, which included age, race, sex, cause of end-stage renal disease (ESRD), dialysis vintage, history of coronary artery disease (defined as previous myocardial infarction or revascularization procedure), hypertension, and diabetes status from the patient’s clinical record. This study was approved by the St Michael’s Hospital Research Ethics Board. As the BIS data for this study were recorded to guide routine patient care, a waiver of patient-level consent was authorized.

#### Anthropometric measurements

Participants were weighed without outdoor clothing and no footwear. Body weight was measured to the nearest 0.1 kg on a TRONIX digital platform scale (TRONIX 5702 Bariatric Stand-On Scale, www.scale-tronix.com). Height was measured to the nearest 0.1 cm using a wall mounted stadiometer (TRONIX 5702) with participants standing erect and arms hanging freely at their sides. Waist circumference was measured at the midpoint between the inferior margin of the last rib and the crest of the ilium with the observer at eye-level to the tape and at the end of a normal expiration.^8^ Midarm circumference was measured at the midpoint between the tip of the shoulder and the tip of the elbow (olecranon process and the acromion). The values were recorded to the nearest 0.1 cm. Skinfold thickness was measured predialysis at 4 sites (biceps, triceps, subscapular, and suprailiac) using a Harpenden skinfold caliper (Baty International RH15 9LR, England). If an arteriovenous fistula or graft was present, biceps and triceps skinfold thickness were measured on the contralateral arm. Skinfold thickness from each location was used to calculate body density (Online Appendix Table 1) which subsequently permitted the calculation of fat mass as described by Durnin and Womersley.^3,9^ All measurements were performed by one observer (N.N.).

### BIS-TBW

We used the Body Composition Monitor (BCM; Fresenius Medical Care, Bad Homburg, Germany) to measure body compartments using BIS. Electrodes were attached to one hand and one foot (in presence of arteriovenous access, the limbs contralateral to the access were used) after a 2- to 3-minute resting period in the supine position before the dialysis session. The following parameters were displayed in liters: TBW, extracellular water, intracellular water, and the extent of overhydration. Overhydration represents the excess fluid and is based upon the fixed proportions of intracellular water and extracellular water within adipose and nonadipose tissue.^7,10^ As BIS was performed before dialysis (to avoid the problem of postdialysis fluid redistribution), we subtracted the ultrafiltration volume during dialysis to calculate the postdialysis TBW (dry weight TBW).

## Clinical Measurements

Laboratory data from within the month prior to the BIS assessment, including hemoglobin, serum albumin, intact parathyroid hormone (PTH), total cholesterol, creatinine, potassium, calcium and phosphorus, were recorded.

Dialysis session data (dialysis session duration, dialysate composition, ultrafiltration volume, relative blood volume changes pre- and post-dialysis systolic and diastolic pressure) were recorded. Kt/V_urea_, as measured by sodium dialysance, was generated by the dialysis machine software using the Watson equation value for determination of “V” (TBW).

## Watson TBW

We calculated TBW in liters using the Watson formula as follows^5^:

$$\begin{array}{l} \mathrm{TBW}\;\left(\mathrm{men}\right)=\;2.447\;+\;0.3362\;\times \;\mathrm{postdialysis weight}\;\left(\mathrm{kg}\right)\;+\;0.1074\;\times \;\mathrm{height}\;\left(\mathrm{cm}\right)\;–\;0.09516\;\times \;\mathrm{age}\\ \mathrm{TBW}\;\left(\mathrm{women}\right)=\;-2.097\;+\;0.2466\;\times \mathrm{postdialysis weight}\;\left(\mathrm{kg}\right)\;+\;0.1069\;\times \mathrm{height}\;\left(\mathrm{cm}\right). \end{array}$$

### Statistical Methods

We used standard descriptive statistics to characterize our study population. We used the Bland-Altman method to calculate the mean difference (the “bias”) and limits of agreement (reference range for difference) between the TBW derived from the Watson formula and the BIS-TBW, which we considered the reference standard for the purpose of this analysis.^11^ The difference between BIS-TBW and Watson-derived TBW was then related to age, waist circumference, and fat mass by using separate regression analyses in males and females. As body composition might be different in individuals of African descent as well as diabetics, we calculated the difference of BIS-TBW and Watson TBW across these subgroups (African descent vs none, diabetics vs nondiabetics, and obese vs normal-weight).

To derive new equations for TBW estimation, Pearson’s correlation coefficients between BIS-TBW (which we considered the reference standard) and other candidate variables were examined. These variables included age, race/ethnicity, body mass index (BMI), diabetes, weight, height, dialysis vintage, waist circumference, and all skinfold thicknesses. As women tend to have a higher percentage of body fat and thus a lower percentage of body water, we made an a priori decision to derive separate equations for men and women. After evaluating univariate relationships, multiple linear regression analyses with stepwise selection was performed to determine variables for the regression equations in our patients. We used the least squares regression analysis to develop the most parsimonious equations for TBW. To examine differences between TBW estimated by our equations and BCM-measured TBW, we used the Bland-Altman method to calculate the mean difference (the “bias”) and limits of agreement (reference range for difference). Statistical analyses were carried out with STATA statistical software version 11.0 (Stata Corporation, www.stata.com).

## Results

### Patient Characteristics

After identifying 203 patients who met our inclusion criteria, we excluded 14 patients with limb amputations, 2 who did not agree to have skinfold measurements, and 3 who refused BIS assessment. Our analytic population comprises 184 patients whose characteristics are summarized in Table 1.

**Table 1.** Characteristics of Patient Population.

	Total cohort (n = 184)	Male (n = 109)	Female (n = 75)
Age (years)	64 ± 15	64 ± 14	64 ± 15
Cause of ESRD			
Diabetes (%)	36	36	36
Hypertension (%)	6	4	10
Glomerulonephritis (%)	27	23	32
History of coronary artery disease (%)	33	38	26
History of diabetes (%)	52	56	45
History of hypertension (%)	84	86	80
Median time on dialysis, years (IQR)	5.0 (2.0-8.1)	4.0 (2.0-8.0)	5.0 (2.5-10.0)
Race			
Caucasian (%)	39	43	33
Black (%)	21	18	27
Asian^a^ (%)	21	18	27
South Asian^b^ (%)	17	21	12
Other (%)	1	1	1
Postdialysis weight (kg)	70.1 ± 16.5	73.7 ± 15.7	64.9 ± 16.3
Body mass index (kg/m^2^)	27.0 ± 5.4	27.1 ± 5.3	26.7 ± 5.6
Kt/V_urea_^c^	1.50 ± 0.36	1.40 ± 0.33	1.64 ± 0.34
Body fat percent by BIS	37.6 ± 10.2	35.5 ± 10.1	40.6 ± 9.5
Fat free mass percent by BIS	62.4 ± 10.1	64.5 ± 10.1	59.4 ± 9.5
Laboratory measurements			
Blood hemoglobin (g/L)	105.3 ± 13.0	105.9 ± 13.2	104.4 ± 12.8
Serum albumin (g/L)	38.0 ± 4.4	38.2 ± 4.7	37.7 ± 3.9
Serum creatinine (µmol/L)	749 ± 320	775 ± 345	709 ± 278
Total cholesterol (mmol/L)	3.73 ± 1.02	3.66 ± 1.06	3.84 ± 0.96
LDL cholesterol (mmol/L)	1.89 ± 0.83	1.88 ± 0.86	1.90 ± 0.77
Serum calcium (mmol/L)	2.21 ± 0.25	2.21 ± 0.28	2.22 ± 0.23
Serum phosphorus (mmol/L)	1.54 ± 0.62	1.46 ± 0.48	1.65 ± 0.77
Serum parathyroid hormone (pmol/L)	43.5 ± 22.8	41.0 ± 23.2	46.3 ± 22.2
Serum potassium (mmol/L)	4.7 ± 0.9	4.6 ± 0.8	4.7 ± 1.0
Predialysis serum urea (mmol/L)	21.8 ± 6.9	22.0 ± 7.5	21.5 ± 5.7

*Note.* ESRD = end-stage renal disease; BIS = bioimpedance spectroscopy; IQR = interquartile range; LDL= low density lipoprotein.

a Asian; Chinese, Japanese, Korean, Filipino, Laotian, Vietnamese.

b South Asian; Indian, Indo-Caribbean, Pakistani, Sri Lankan.

c Kt/V_urea_ was measured using sodium dialysance during dialysis where V was based on the Watson formula.

### Correlation Between Watson Formula–Derived TBW and BIS-TBW

TBW values based on the Watson method had a high correlation with BIS-TBW (correlation coefficients = 0.87 and *P* < .001) (Table 2). Despite the high correlation, the Watson formula overestimated TBW by 4.6 (95% confidence interval [CI]: 4.1-5.1) liters. This overestimation was consistent in predefined categories based on sex, diabetes mellitus status, race, and BMI (Table 2).

**Table 2.** Correlation, Bias, and Limits of Agreement of Watson TBW and BIS-TBW in Total Population and in Different Subgroups.

Population	BIS-TBW (L)	Watson TBW (L)	Correlation (*r*)^a^	Bias (95% CI) (L)	LOA (L)
All (n = 184)	31.1 ± 6.5	35.7 ± 7.0	0.87*	4.6 (4.1-5.1)	−2.5 to 11.6
Male (n = 109)	33.8 ± 6.0	38.9 ± 6.2	0.83*	5.1 (4.5-5.8)	−1.9 to 12.2
Female (n = 75)	27.2 ± 5.4	31.0 ± 5.3	0.80*	3.8 (3.0-4.5)	−3.0 to 10.5
Nondiabetes (n = 89)	30.7 ± 6.6	35.4 ± 7.0	0.86*	4.6 (3.8-5.4)	−2.6 to 11.9
Diabetes (n = 95)	31.4 ± 6.6	35.9 ± 7.0	0.88*	4.5 (3.8-5.2)	−2.3 to 11.3
Black (n = 39)	32.6 ± 5.6	37.0 ± 5.8	0.70*	4.4 (2.9-5.8)	−4.5 to 13.2
Nonblack (n = 145)	30.7 ± 6.8	35.4 ± 7.3	0.89*	4.6 (4.1-5.2)	−1.9 to 11.1
BMI > 30 (n = 50)	35.2 ± 6.5	41.7 ± 6.2	0.84*	6.4 (5.4-7.5)	−0.9 to 13.8
BMI ≤ 30 (n = 134)	29.6 ± 5.9	33.5 ± 6.0	0.85*	4.6 (3.8-5.4)	−2.5 to 10.3

*Note.* TBW = total body water; BIS = bioimpedance spectroscopy; CI = confidence interval; LOA = limits of agreement (total range of difference of Watson with BIS-TBW); BMI = body mass index.

a Values are correlation coefficients (*r*).

* *P* < .001.

**Figure 1.**
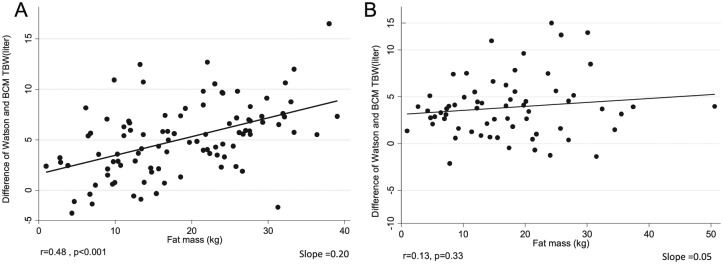
The effect of fat mass (calculated based on Durnin and Womersley formula) on the variation between Watson and BIS-TBW in 109 men (A) and 75 women (B). *Note.* BIS = bioimpedance spectroscopy; TBW = total body water.

**Figure 2.**
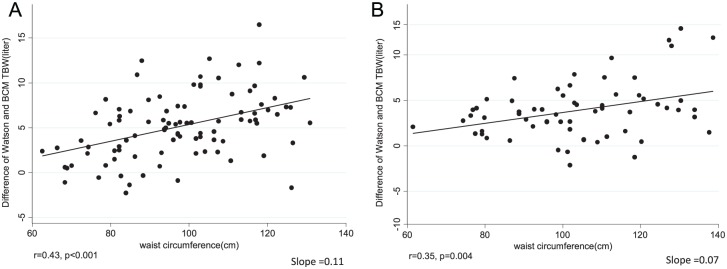
The effect of waist circumference on the variation between Watson and BIS-TBW in 109 men (A) and 75 women (B). *Note.* BIS = bioimpedance spectroscopy; TBW = total body water.

We examined the association between patient characteristics and the extent of TBW overestimation by the Watson formula. Figures 1 and 2 examine this issue by plotting the magnitude of overestimation of TBW by the patient’s waist circumference and body fat mass calculated from skinfold thicknesses. In men, both higher total fat mass (general obesity) and higher waist circumference (abdominal obesity) were correlated with TBW overestimation when using the Watson formula. The correlation coefficients between fat mass and waist circumference, respectively, with TBW overestimation were 0.48 and 0.43 (*P* < .001 for both correlations). In women, higher abdominal obesity, but not general obesity, was associated with higher TBW overestimation by the Watson formula. Age was not associated with TBW overestimation with the Watson formula in both men and women (data not shown).

### The Association Between Different Approaches to Kt/V_urea_ Estimation

**Figure 3.**
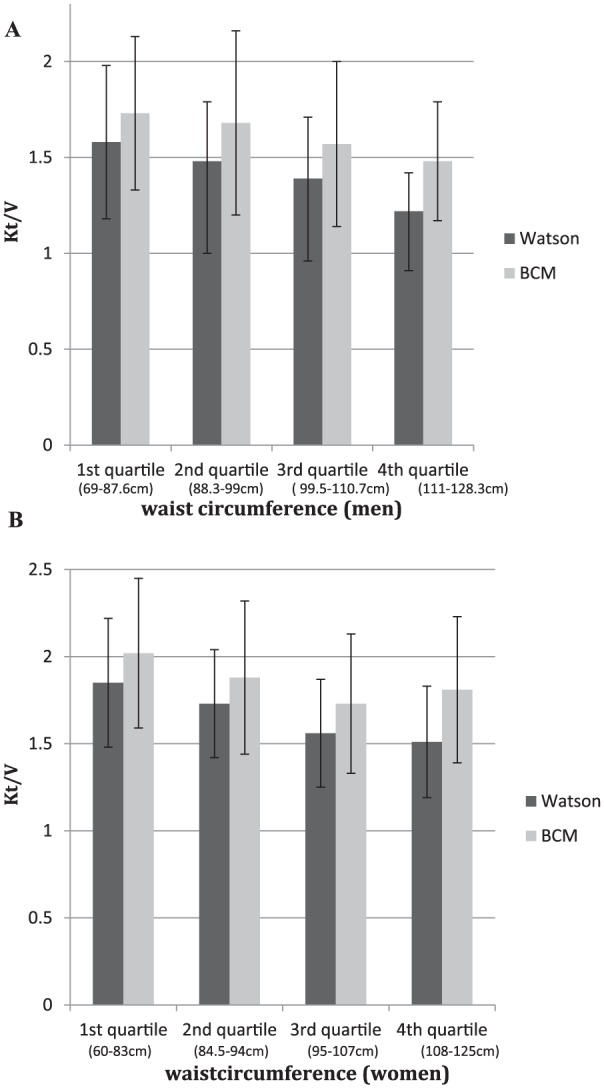
Kt/V_urea_ calculated by Watson versus BIS across quartiles of waist circumference in 109 men (A) and 75 women (B). *Note.* BIS = bioimpedance spectroscopy; BCM = Body Composition Monitor.

In Figure 3, we compared Kt/V_urea_ measures in which the V component was calculated by the Watson formula and BIS across quartiles of waist circumference. Application of the Watson formula underestimated Kt/V_urea_ in all 4 quartiles, and most prominently in patients in the highest waist circumference quartile.

### Derivation of a Novel Equation for TBW Estimation

We used multiple linear regression to derive equations that predict BIS-measured TBW. Stepwise procedures led to the selection of 2 variables (weight and waist circumference) in both men and women (root mean square error [RMSE]: 3.48 and 2.84, respectively]:

Our derived equations are as follows:

$$\begin{array}{l} {\mathrm{TBW}}_{\mathrm{men}}=\;25.67450+\;0.\;5880\times \;\mathrm{weight}\;\left(\mathrm{kg}\right)\;-\;0.\;3556\times \mathrm{waist circumference}\;\left(\mathrm{cm}\right)\\ {\mathrm{TBW}}_{\mathrm{women}}=\;17.6071+0.\;3823\times \;\mathrm{weight}\;\left(\mathrm{kg}\right)\;-\;0.\;1573\times \;\mathrm{waist circumference}\;\left(\mathrm{cm}\right). \end{array}$$

TBW values based on our equations had a high correlation with BIS-TBW (correlation coefficients = 0.86 and 0.85 for men and women respectively, *P* < .001; Table 3). As compared with the Watson formula–derived TBW, TBW as predicted by our equations had lower bias and higher limits of agreement with BIS-TBW (Table 2)

**Table 3.** Correlation, Bias, and Limits of Agreement of BCM-TBW and Our Equations TBW in Men and Women.

Population	BIS-TBW (L)	Our equations TBW (L)	Correlation (*r*)^a^	Bias (95% CI) (L)	LOA (L)
Male (n = 109)	33.8 ± 6.0	33.9 ± 4.8	0.86*	−0.001 (–0.72 to 0.72)	−6.9 to 6.9
Female (n = 75)	27.2 ± 5.4	27.4 ± 4.4	0.85*	0.004 (–0.70 to 0.71)	−5.6 to 5.6

*Note.* BIS = bioimpedance spectroscopy; TBW = total body water; CI = confidence interval; LOA = limits of agreement (total range of difference of Watson with BIS-TBW).

a Values are correlation coefficients (*r*).

* *P* < .001.

**Figure 4.**
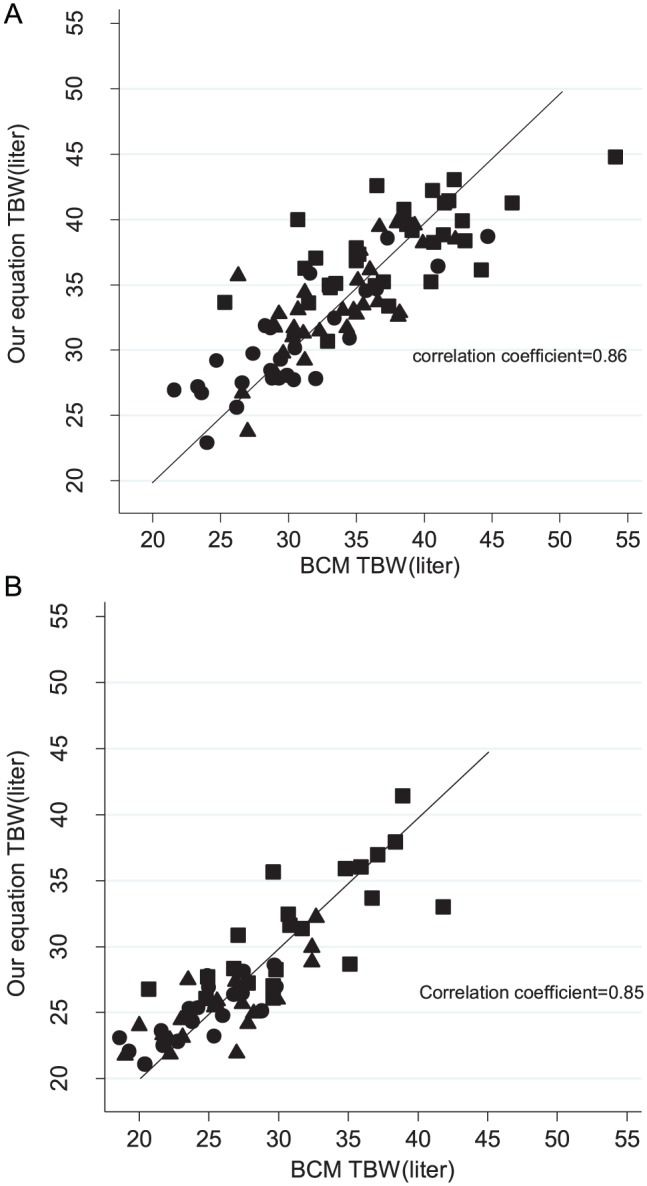
Correlation of our proposed equations for TBW with the BIS-measured TBW across tertiles of waist circumference in 109 men (A) and 75 women (B). *Note.* Circles: first tertile of waist circumference; triangles: second tertile of waist circumference; squares: third tertile of waist circumference. Line displays line of identity. TBW = total body water; BIS = bioimpedance spectroscopy; BCM = Body Composition Monitor.

In Figure 4, we evaluated the agreement between our equations’ estimate of TBW and the BIS-TBW across tertiles of waist circumference. TBW estimation readily approximated BIS measurements and discrepancies were unaffected by waist circumference.

## Discussion

In a contemporary cohort of dialysis recipients, we found that TBW, a vital element in the estimation of Kt/V and dialysis adequacy, is consistently overestimated when the Watson formula is employed. Although the Watson formula is the recommended tool for TBW estimation, it provides a consistently inaccurate assessment of TBW which in turn leads to an underestimation of Kt/V_urea_ in most patients. Using BIS-derived TBW as a reference standard and thorough evaluation of body composition using comprehensive anthropometric measurements, we developed 2 practical estimating equations for TBW. Pending further validation, these equations will be potentially useful in units where bioimpedance technology is not available.

Watson et al initially derived their formula from a cohort of 458 adult males and 265 adult females in whom the reference standard for TBW was measured using traditional dilution methods (known amounts of diluents such as deuterium or tritium oxide or antipyrine, which diffuse freely through all body compartments with no permeability barrier).^5^ This resulted in a readily applicable equation that comprised height, weight, and age.^5^ Although not a single dialysis recipient was included in the derivation cohort for the Watson formula, HD guidelines recommend applying this formula in the dialysis population.^2^ This is problematic because in dialysis patients, weight and height measurements do not necessarily reflect the individual’s relative proportion of fat and muscle (the proportion of water in lean and adipose tissue is 70% and 20%, respectively).^7^ Furthermore, muscle wasting and obesity are frequent in the dialysis population.^12,13^

Our work suggests that waist circumference, a marker of abdominal fat, was more effective in predicting TBW and in our derived equations, supplanted height, which is a key element of the Watson formula. This might be due to the fact that TBW depends on the relative proportion of fat and muscle, which is better captured by waist circumference (and weight) than by height.

Chertow et al used single-frequency bioimpedance to estimate TBW and derived a novel equation for TBW estimation in HD patients.^4^ We calculated the TBW in our population using the Chertow method, but it also overestimated TBW, especially in obese individuals. Differences in bioimpedance technologies that were used to determine TBW in the Chertow study might explain the differences between the estimated TBW generated by that equation and the BIS readings. It has since been shown that BIS predicts TBW with better accuracy than single-frequency bioimpedance.^4,14^ BIS measures body fluid at 50 frequencies, and while high-frequency current passes through the TBW, low-frequency current cannot penetrate cell membranes and thus flows exclusively through the extracellular water.^7^ Therefore, BIS determines the electrical resistances of the TBW and the extracellular water and enables clear separation between extracellular and intracellular water by the extremely wide range of measurement frequencies. BIS has been validated against relevant gold standard measures in both healthy individuals and in chronic dialysis patients to assess hydration status.^10,15^ These standard measures were sodium bromide for extracellular water, deuterium for TBW, and body potassium for intracellular water.^16^ Comparison of BIS against these reference standards showed excellent concordance; therefore, we feel confident in our decision to treat BIS-measured TBW as the reference standard.

Although previous studies have demonstrated that the Watson equation overestimates the TBW in HD patients (Table 4),^17-21^ a unique feature of our study is the comprehensive anthropometric assessments to help explain the reasons behind this finding. Lee et al developed equations in men and women to estimate TBW.^17^ We compared the TBW based on their equations and ours and found that there was a high correlation between their TBW estimates and BIS. Similar to the Watson and Chertow equations, however, their equation overestimated TBW in men with higher fat mass. Furthermore, the technology they used was segmental bioelectrical impedance analysis as compared with multifrequency BIS that has a better theoretical foundation and has been validated as a technique to assess TBW.^10^ Also they did not perform comprehensive anthropometric evaluation including 4-site skinfold thicknesses and waist circumference. Another study which used BIS also concluded that body composition affects TBW estimated by Watson TBW and Kt/V_urea_ is underestimated with Watson formula.^21^ Although BIS can determine fat and muscle the problem with using only the BIS as a measure of fat and muscle is that BIS calculates these components based on the amount of TBW (difference in the hydration parameters of adipose tissue and nonadipose tissue) and not independently.

**Table 4.** Summary of Studies Evaluating Accuracy of Anthropometric TBW Measurement in HD Patients.

Study	Patient	Reference method	Conclusions
Lee et al 2001^17^	101 HD	Bioelectrical impedance	Currently available TBW-estimating equations (Watson, Hume-Weyers, and Chertow) overestimate TBW in HD patients.
Kloppenburg et al 2001^18^	54 HD	Direct dialysate quantitation	Anthropometry-based equations (Watson equations, a fixed proportion of postdialysis body weight and skinfold thickness measurements) overestimate TBW in HD patients, leading to an overestimation of PNA values.
Daugirdas et al 2003^20^	1124 HD	Urea kinetic models	Commonly used equations to predict the TBW (Watson, Hume-Weyers, and Chertow) markedly overestimate the urea distribution volume in HD patients.
Davenport 2013^19^	363 HD	Multifrequency bioelectrical impedance analysis	Prescribing dialysis based on the Watson equation leads to underestimation of Kt/V_urea_ in obese patients.
Vega et al 2015^21^	144 HD	Bioimpedance spectroscopy	Body composition affects TBW estimated by V Watson and in young patients who present more lean tissue and less fat tissue. Kt/V_urea_ is underestimated with Watson TBW.

*Note.* TBW = total body water; HD = hemodialysis; PNA = protein nitrogen appearance.

The traditional reliance on the Watson equation to estimate TBW leads to a consistent underestimation of Kt/V_urea_ in most patients. In centers where minimum Kt/V targets determine dialysis prescriptions, this may result in unnecessary increases in dialysis exposure which may negatively impact on the patient’s quality of life. On the other hand, we found a small number of patients (< 10% of our cohort), generally those with a lower waist circumference, in whom the Watson formula underestimated TBW as compared with the BCM-derived TBW. In such cases, there would be an overestimation of the Kt/V_urea_ and perhaps such patients might be underdialyzed.

Our data emphasize the importance of measuring waist circumference as a major component of the anthropometric estimating equations. Although body composition is known to change with age, with a reduction in muscle mass and increase in fat mass, unlike the Watson formula, age was not a significant predictor in our equations.^22^ Our equations suggest that in both men and women, the combination of waist circumference plus weight is the best surrogate of fat/muscle contribution and so should be utilized to predict TBW. As the greatest overestimation of Kt/V by Watson formula exists in the patients with highest waist circumference, Kt/V estimation, by the Watson formula, should be interpreted with heightened caution in these patients.

The principal strength of this study was the performance of a comprehensive anthropometric evaluation that included waist circumference (as a surrogate of abdominal fat) and midarm circumference plus 4 measures of skinfold thickness (as surrogates of fat and muscle in different parts of the body), which to the best of our knowledge has not been done in any other study that has assessed TBW in the dialysis population. This allowed us to identify waist circumference as a more robust predictor of TBW than height. The current study also used BIS, the most accurate bioimpedance technology available, which has been validated against gold-standard methods in calculating TBW.^15^ Finally, we developed straightforward equations for TBW estimation that, once validated, can be readily applied in settings where bioimpedance is not readily available.

Our study has evident limitations. First, we did not perform an external validation of our derived equations. Thus, our equations will not be ready for widespread application until they are validated in other patient populations. A further shortcoming is our assumption that BIS-measured values are the reference standard for TBW. Although BIS has been shown to be robust when compared with classic standards for body composition assessments, it may be premature to treat it as the true “gold standard.” The sample size was modest, and the size of certain subgroups (eg, those of African descent) was small thereby limiting our ability to explore polynomial and multiplicative interaction terms.

Although the urea reduction ratio (URR, which is calculated as the difference between the predialysis and postdialysis urea concentration/predialysis urea concentration) provides a convenient assessment of urea removal in a given HD session and does not require an assessment of TBW, we did not collect pre- and post-dialysis urea samples for the sessions on which BCM-TBW was assessed.^22^ Thus, we cannot comment on the extent to which Kt/V_urea_ values using V from our equations compare with the URR. However, the benefit of an accurate Kt/V provided by the dialysis machine is that it is available for every treatment, not only when blood is drawn. Furthermore, beyond its application in the Kt/V calculation, accurate estimation of the TBW can provide insight into the volume of distribution of drugs administered to dialysis patients, and provides an accurate estimate of TBW for the correction of acute hypernatremia and hyponatremia in hemodialysis patients.

## Conclusions

In conclusion, we have shown that the Watson formula provides a gross overestimation of TBW in chronic hemodialysis recipients. As the assessment of small solute clearance is highly affected by TBW estimation, this may lead to clinicians acquiring a distorted perception of a patient’s dialysis adequacy. Measurement of TBW using contemporary bioimpedance technology has the potential to mitigate this problem. Similarly, the estimation of TBW using our novel equations, if validated through ongoing research, might provide clinicians with a more refined assessment of TBW and thus help guide appropriate adjustments to the dialysis prescription. However, until then, these equations should not be applied clinically.
